# Hexa-Longin domain scaffolds for inter-Rab signalling

**DOI:** 10.1093/bioinformatics/btz739

**Published:** 2019-09-28

**Authors:** Luis Sanchez-Pulido, Chris P Ponting

**Affiliations:** Medical Research Council Human Genetics Unit, IGMM, University of Edinburgh, Edinburgh EH4 2XU, UK

## Abstract

**Summary:**

CPLANE is a protein complex required for assembly and maintenance of primary cilia. It contains several proteins, such as INTU, FUZ, WDPCP, JBTS17 and RSG1 (REM2- and RAB-like small GTPase 1), whose genes are mutated in ciliopathies. Using two contrasting evolutionary analyses, coevolution-based contact prediction and sequence conservation, we first identified the INTU/FUZ heterodimer as a novel member of homologous HerMon (Hermansky-Pudlak syndrome and MON1-CCZ1) complexes. Subsequently, we identified homologous Longin domains that are triplicated in each of these six proteins (MON1A, CCZ1, HPS1, HPS4, INTU and FUZ). HerMon complexes are known to be Rab effectors and Rab GEFs (Guanine nucleotide Exchange Factors) that regulate vesicular trafficking. Consequently, INTU/FUZ, their homologous complex, is likely to act as a GEF during activation of Rab GTPases involved in ciliogenesis.

**Supplementary information:**

Supplementary data are available at *Bioinformatics* online.

## 1 Introduction

Many diverse cell processes are regulated by small GTPases, switching between active (GTP-bound) and inactive (GDP-bound) states. Small GTPases are switched on by guanine-nucleotide exchange factors (GEFs) that promote the exchange of bound GDP by GTP (Bourne *et al.*, 1990). Mutations in small GTPases and GEFs are frequent in Mendelian diseases and cancer (Blacque *et al.*, 2018; Bos *et al.*, 2007). Multiple small GTPases of the Rab family and their GEFs, for example, are critical for the assembly of cilia (ciliogenesis) and can be mutated in ciliopathies (Blacque *et al.*, 2018).

Mouse mutants for genes encoding the Rab-like small GTPase RSG1 or ciliopathy-associated proteins Fuzzy (FUZ) and Inturned (INTU) show developmental abnormalities characteristic of decreased cilia-dependent Hedgehog signalling (Agbu *et al.*, 2018; Gray *et al.*, 2009; Zeng *et al.*, 2010). These three proteins interact as members of the ciliogenesis and planar polarity effector (CPLANE) complex that controls recruitment of intraflagellar transport machinery to the basal body (Toriyama *et al.*, 2016). The precise molecular and cellular roles in ciliogenesis of these proteins remain unknown. This is in large part, it is proposed, because they lack discernible domain homologues (Adler and Wallingford, 2017). INTU protein is a scaffolding subunit of the CPLANE complex and, with the sole exception of a PDZ (PSD-95/discs large/ZO-1) domain, no other functional domain has been identified within its 942 residues length (Adler and Wallingford, 2017; Chang *et al.*, 2015; Toriyama *et al.*, 2016; Wang *et al.*, 2018; Yang *et al.*, 2017). To investigate the evolutionary provenance of the INTU protein family, we embarked on a deep sequence analysis taking advantage of both protein sequence conservation and coevolution-based contact prediction approaches.

## 2 Results and discussion

### 2.1 A new Longin domain in INTU

**Fig. 1.**
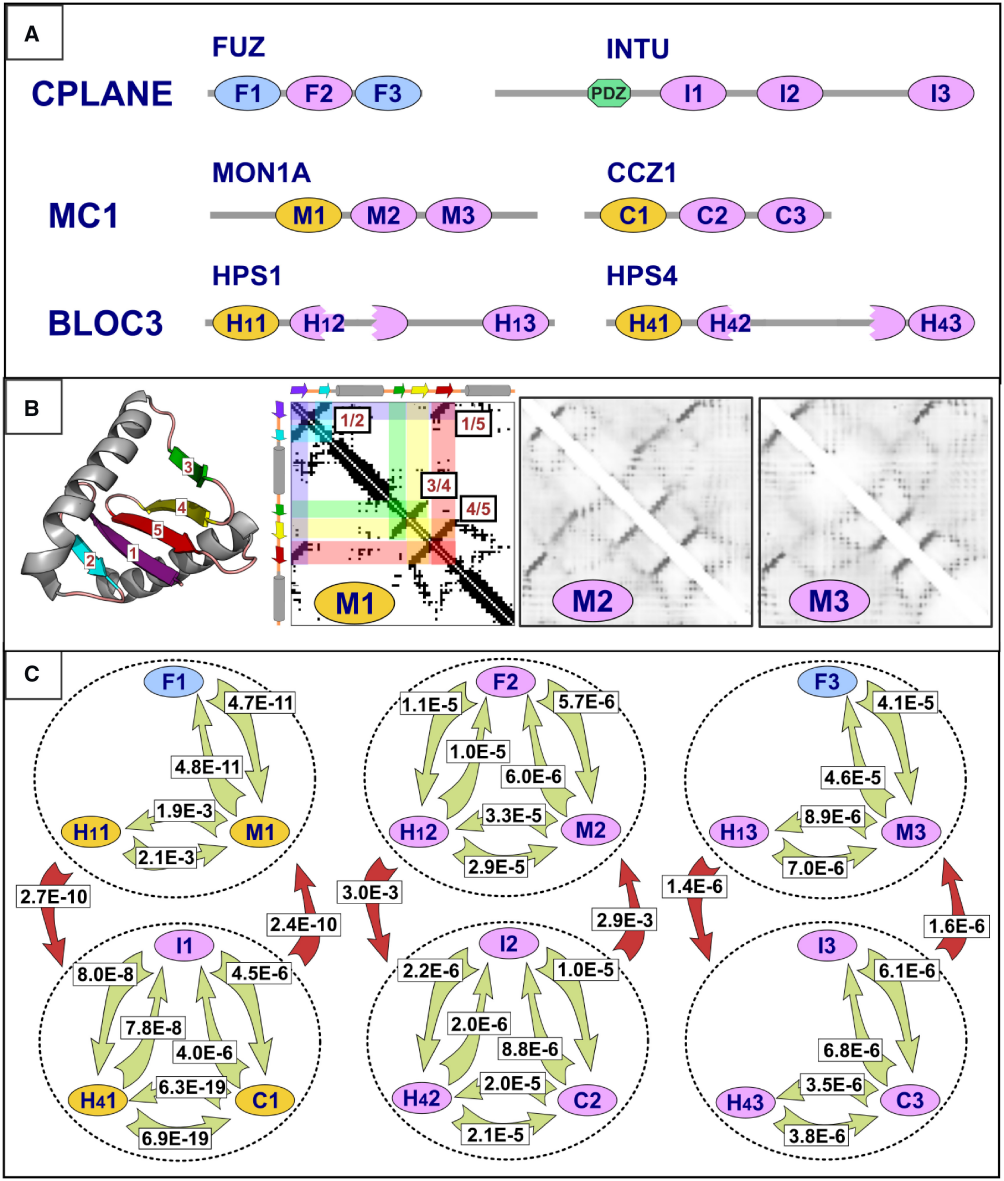
(**A**) HerMon family domain architecture. Domains coloured in gold are the Longin domains identified first by Kinch and Grishin in MON1A, CCZ1, HPS1 and HPS4 (ovals labelled M1, C1, H11 and H41) (Kinch and Grishin, 2006). The similarity between the N-terminal regions of Ccz1 and Hps4 was originally found by Hoffman-Sommer *et al.* (Hoffman-Sommer *et al.*, 2005) and termed the CHiPS domain, corresponding to Longin domains labelled C1 and H41. The first and third Longin FUZ domains (F1 and F3; coloured in blue) were previously proposed, without statistical evidence from sequence similarities, using the GenTHREADER method of structure prediction (Gray *et al.*, 2009; Lobley *et al.*, 2009; Toriyama *et al.*, 2016). In the second Longin domains of HPS1 and HPS4 there are long insertions showing poor evolutionary conservation (H12 and H42; broken ovals). The PDZ domain of INTU annotated in the SMART domain database (hexagon coloured in green) (Letunic and Bork, 2018). Newly identified Longin domains are shown in red (F2, I1, I2, I3, M2, M3, C2, C3, H12, H13, H42 and H43). (**B**) MON1A contact maps. Cartoon of the Longin domain structure of *C. thermophilum* MON1 (PDB: 5LDD_A; amino acids 222-316) core structure (β-strands are labelled 1 to 5 and coloured in purple, cyan, green, yellow and red, respectively) generated using PyMOL (https://pymol.org/). Anti-parallel β-strand pairs are clearly observable in the contact map calculated from the first Longin domain (M1) of *C.thermophilum* MON1 (PDB: 5LDD_A) (see β-strand pairs 1/2, 1/5, 3/4 and 4/5), whose structure is known (Kiontke *et al.*, 2017), generated using the Cocomaps server (input: 5LDD_A versus 5LDD_A, cut-off distance value = 7 Ångstroms) (Vangone *et al.*, 2011). Two similar contact patterns, predicted with RaptorX (Wang *et al.*, 2017), are observed in two conserved regions in human MON1A protein (M2 and M3, amino acids 316-415 and 444-544, respectively) (Supplementary Figs S3, S10 and S12). (**C**) HHpred comparison E-values among pairs of HerMon Longin domains. Numbers overlaid onto green arrows correspond to HHpred profile-versus-profile comparison Erpt-values (Söding *et al.*, 2005). Erpt is the estimated number of alignments with a particular score, or higher, in a reduced search space of 18 Longin domain profiles (those shown in panel A) (Söding *et al.*, 2005) and indicates the significance of profile-profile alignment scores conditional to these proteins harbouring at least one Longin domain. Numbers overlaid on red arrows correspond to HHpred profile-versus-profile comparison Erpt -values of 6 profiles that each represents each of the three Longin domains in FUZ, MON1A and HPS1, or in INTU, CCZ1 and HPS4 (indicated within circles with dotted lines). Multiple sequence alignments, on which these profiles are based, are provided in Supplementary Figures S1, S2 and S9–S12. Arrows indicate the profile search direction. Only E-values < 0.005 are shown

We initiated our analyses with a JackHMMER iterative search (Finn *et al.*, 2015) of the UniRef50 database (The UniProt Consortium, 2019) using the human INTU protein sequence as query. This identified full-length INTU homologues across the animal kingdom. A full-length multiple sequence alignment, generated with T-Coffee (Notredame *et al.*, 2000), revealed an evolutionarily conserved region (INTU_HUMAN amino acids 305–439) just after its PDZ domain (Fig. 1).

HHpred searches against the PDB70 profile database (Söding *et al.,* 2005) using this conserved region as input detected significant sequence similarity with the Longin domain from *Chaetomium thermophilum* CCZ1 (Kiontke *et al.*, 2017) with an E-value of 0.01 (Probability: 95.4). Moreover, in support of this top match, the next most statistically significant similarities were with additional members of the Longin superfamily. Furthermore, the predicted secondary structure (Jones, 1999) of this INTU conserved region was consistent with known Longin domain structures (Supplementary Fig. S1).

Longin domains were described initially as evolutionarily conserved N-terminal regions of VAMP7 (Vesicle-associated membrane protein 7) and Ykt6 protein families (Filippini *et al.*, 2001). Structural analysis subsequently identified similarities between two AP2 (adaptor protein 2) complex subunits (AP2A2 (subunit alpha-2) and AP2M1 (subunit mu)) and Sec22b protein and Ykt6 Longin domain (Collins *et al.*, 2002). Longin domains have since been found widely across eukaryotes (Pfam family Longin, accession: PF13774; InterPro, accession: IPR011012) (Mitchell *et al.*, 2019; Punta *et al.*, 2012) and have often been implicated in aspects of membrane dynamics regulation (Daste *et al.*, 2015). In structural terms, the Longin core, and its related roadblock fold, are composed of an α/β fold containing two α-helices organized around a central β-sheet of five anti-parallel β-strands (Fig. 1) (Kinch and Grishin, 2006; Kiontke *et al.*, 2017; Levine *et al.*, 2013).

These similarities in primary sequence and secondary structure correspondence indicate that INTU is a previously undescribed member of the Longin domain-containing protein family. We were struck by INTU's interacting partner FUZ also containing a proposed N-terminal Longin domain (Toriyama *et al.*, 2016) because Longin domains commonly heterodimerise with other Longin domains (Kinch and Grishin, 2006; Kiontke *et al.*, 2017; Levine *et al.*, 2013).

Consequently, we decided to further analyze this putative N-terminal Longin domain in FUZ (amino acids 10-141) and found it to have statistically significant sequence similarity to the Longin domain of *C. thermophilum* MON1 (HHpred E-value = 1.7x10^−8^; Probability: 98.6) (Kiontke *et al.*, 2017) (Supplementary Fig. S2). This pair of N-terminal INTU/FUZ Longin domains were thus strikingly each found, by HHpred searches, to be homologues of the pair of Longin domains that heterodimerise in CCZ1 and MON1, respectively.

Our analysis thus indicates that INTU/FUZ is a third and unanticipated, heterodimer of the HerMon family that was previously represented by only MC1 (MON1/CCZ1 heterodimer) and BLOC3 (Biogenesis of lysosome-related organelles complex 3) complexes (the latter composed of the HPS1/HPS4 heterodimer) (Supplementary Figs S1 and S2) (Barr, 2014; Carmona-Rivera *et al.*, 2013; Chiang *et al.*, 2003; Gerondopoulos *et al.*,2012; Kiontke *et al.*, 2017; Kinch and Grishin, 2006; Nazarian *et al.*, 2003).

### 2.2 Tandemly repeated Longin domains in the HerMon family

To date, there is structural and/or statistically significant sequence similarity evidence for only a single N-terminal Longin domain within MON1, CCZ1, HPS1 and HPS4 proteins (M1, C1, H11 and H41; indicated in gold in Fig. 1) (Kiontke *et al.*, 2017; Kinch and Grishin, 2006). To identify putative domains in the unassigned C-terminal regions of these four proteins and the INTU/FUZ heterodimer, we took advantage of two distinct types of evolutionary information, namely protein sequence conservation and coevolution-based contact predictions.

Residue pairs in close contact in protein 3D structures often show a correlated mutational signature. This is due to a missense mutation in one residue often being compensated by a missense mutation in its paired residue, so as to preserve protein stability, folding or function (Rollins *et al.*, 2019; Schmiedel and Lehner, 2019). Coevolution-based contact predictions methods are able to identify such mutationally coupled residues across deep multiple sequence alignments (Wang *et al.*, 2017).

Coevolution-based contact predictions using RaptorX (Wang *et al.*, 2017) revealed a repeated contact pattern, observed three-times in each of MON1, CCZ1, INTU and FUZ HerMon family members (Fig. 1; Supplementary Figs S3–S6). This common pattern then allowed us to define the boundaries delimiting three repeated regions. In MON1 and CCZ1 the first of these regions correspond to their structurally determined Longin domains (Kiontke *et al.*, 2017). In particular, their longer β-strands 1 and 5, buried within the structural core of the Longin fold, contribute a strong feature of the triplicated contact pattern (Fig. 1B;Supplementary Figs S3 and S4).

This repeated contact pattern was not evident for HPS1 and HPS4 (Supplementary Figs S7 and S8), likely owing to the limited phyletic range, and thus sequence divergence, within these families. Even the previously identified N-terminal Longin domains in HPS1 and HPS4 (Kinch and Grishin, 2006), and confirmed by us (Supplementary Figs S1 and S2), are not apparent from these contact prediction maps (Supplementary Figs S7 and S8). Similarities between HPS1 or HPS4, and MON1 or CCZ1, respectively, were observed from detailed sequence analysis (Fig. 1; Supplementary Figs S9–S12).

These findings, based on sequence conservation and coevolution-based contact predictions, led us to a hypothesis that each of these triplicated regions contains a Longin domain, and motivated us to generate 18 multiple protein sequence alignments and profiles, three for each of the six HerMon family proteins: MON1, CCZ1, INTU, FUZ, HPS1 and HPS4 (Supplementary Figs S1, S2 and S9–S12).

Subsequent pairwise comparison of sequence conservation among these profiles using HHpred (Söding *et al.*, 2005) yielded statistically significant sequence similarities among these repeated regions (E < 5.0x10^−3^; Fig. 1; Supplementary Figs S1, S2 and S9–S12) that are indicative of homology.

Consistent with homology, 3 D models generated using RaptorX (Wang *et al.*, 2017) for the second and third MON1 repeats are each consistent with a Longin fold. The highest ranked RaptorX models for the second and third repeats were significantly similar to Longin structures (DALI scores of Z = 6.0 and 7.7, respectively, exceeding the Z-score = 2 threshold for statistical significance) (Holm and Laakso, 2016).

The common triplicated Longin domain architecture of HerMon proteins (Fig. 1) indicates that these 6 proteins diverged from a common ancestral protein pair (MON1/CCZ1 heterodimer), whose evolutionary precursor was a single homodimer containing three consecutively repeated Longin domains.

### 2.3 Functional conservation in HerMon complexes

Homology among the three pairs of HerMon proteins is likely to reflect their similar functional mechanisms. MC1 and BLOC3 complexes are signal transducers in Rab cascades both as effectors of an active Rab (GTP-bound state) (Rab5 for MC1 and Rab9 for BLOC3) and as GEFs of an inactive Rab (GDP-bound state) (Rab7 for MC1 and Rab32/Rab37 for BLOC3) that, consequently, guide the directionality of vesicular traffic to lysosome and lysosome-related organelles (Gerondopoulos *et al.*, 2012; Hegedus *et al.* 2016; Kiontke *et al.*, 2017; Kinchen and Ravichandran, 2010; Kloer *et al.*, 2010; Mahanty *et al.*, 2016; Nordmann *et al.*, 2010; Pfeffer, 2013, 2017). This suggests that the INTU/FUZ heterodimer also orchestrates a Rab signaling cascade in ciliogenesis, involving RAB8 and RSG1, two Rab proteins known to be components of the CPLANE complex (Agbu *et al.*, 2018; Toriyama *et al.*, 2016; Zilber *et al.*, 2013).

## 3 Conclusion

In summary, we have identified the INTU/FUZ heterodimer as the third pair of HerMon heterodimeric complexes and discovered that all six HerMon proteins harbor three Longin domains. Our identification of each HerMon complex as a hexa-Longin domain scaffold should aid in the design of further experiments that investigate their contributions to diverse transport-related processes and inter-Rab signaling pathways.

During proof corrections of this work, Gerondopoulos *et al.* (2019) provided experimental validation of INTU/FUZ as a Rab-GEF complex, and proposed a similar three Longin domain structures without presenting alignments or statistical results.

## Funding

This work was supported by the Medical Research Council UK. 

*Conflict of Interest*: none declared.

## Supplementary Material

btz739_Supplementary_Data
