# Supplementary material for: Hexa-Longin domain scaffolds for inter-Rab signalling
Source: Bioinformatics. 2019 Sep 28;36(4):990–3. doi: 10.1093/bioinformatics/btz739 (PMC7703760; doi:10.1093/bioinformatics/btz739)
Supplement: btz739_Supplementary_Data [file btz739_supplementary_data.zip › SanchezPulidoSupplementaryInfo.pdf]

Supplementary figure legends

Supplementary Figure S1 - First Longin domain of INTU/CCZ1/HPS4.

Alignments were produced with T-Coffee, HMMer (when collecting orthologous proteins and expanding alignments in each family), and HHalign from HH-suite (when comparing and aligning remotely related domains) (Finn *et al.*, 2015; Notredame *et al.*, 2000; Söding *et al.*, 2005) using default parameters, and were refined manually and viewed with the Belvu program (http://sonnhammer.sbc.su.se/Belvu.html). Limits of protein sequences included in the alignment are indicated by flanking residue positions. Numbers shown in green represent amino acids that have been excised from the alignment. The amino acid colouring scheme indicates average BLOSUM62 scores (which are correlated with amino acid conservation) for each alignment column: red (greater than 2.5), violet (between 2.5 and 1) and light yellow (between 1 and 0.3). Sequences are named according to their UniProt identifiers (The UniProt Consortium, 2019). PsiPred secondary structure predictions (Jones, 1999) (with E indicating a β-strand (in red) and H an α-helix (in green)) are shown below the alignment for each family repeat. These predicted secondary structures are consistent with X-ray determined secondary structures of the first Longin domains in CCZ1 and MON1 (Kiontke *et al.*, 2017), labelled with their Protein Data Bank identifier 5LDD, chains B and A, respectively, and shown in first Longin repeats alignments of INTU/CCZ1/HPS4 and FUZ/MON1A/HPS1 families. HerMon family members are indicated by coloured bars to the left of each alignment in red (INTU family), yellow (CCZ1 family), and purple (HPS4 family) in INTU/CCZ1/HPS4 repeats' alignments; and green (FUZ family), orange (MON1 family), and blue (HPS1 family) in FUZ/MON1/HPS1 repeats' alignments. Seed alignments for each of these longin domains have been deposited in PFAM database (ID:Intu_longin_1, PF19031; ID:Intu_longin_2, PF19032; ID:Intu_longin_3, PF19033; ID:Fuz_longin_1,PF19028; ID:Fuz_longin_2 PF19029; ID:Fuz_longin_3, PF19030).

Supplementary Figure S2. First Longin domain of FUZ/MON1/HPS1.

Details are provided in the legend to Supplementary Figure S1. Numbering of Q86VX9_MON1A_HUMAN protein corresponds to its isoform 5.

Supplementary Figure S3. MON1A contact-map.

RaptorX coevolution-based contact prediction, using default parameters, for full-length human MON1A protein (Wang *et al.*, 2017). PsiPred secondary structure predictions (Jones, 1999) for MON1A are shown above the contact map. A triplicated 2D and contact pattern is observed, labelled M1, M2 and M3. For each of these MON1A Longin repeats their β-strands 1 to 5 are coloured in purple, cyan, green, yellow, and red, respectively. The predicted anti-parallel β-strand pairs in each Longin repeat, are easily recognisable from their elevated contact density patterns in the contact map (see β-strand pairs 1/2, 1/5, 3/4, and 4/5).

Supplementary Figure S4. CCZ1 contact-map.

RaptorX coevolution-based contact prediction, using default parameters, for full-length human CCZ1 protein (Wang *et al.*, 2017). PsiPred secondary structure predictions (Jones, 1999) for CCZ1 are shown above the contact map. A triplicated 2D and contact pattern is observed, labelled C1, C2 and C3. For each of these CCZ1 Longin repeats their β-strands 1 to 5 are coloured in purple, cyan, green, yellow, and red, respectively. The predicted anti-parallel β-strand pairs in each Longin repeat, are easily recognisable from their elevated contact density patterns in the contact map (see β-strand pairs 1/2, 1/5, 3/4, and 4/5).

Supplementary Figure S5. FUZ contact-map.

RaptorX coevolution-based contact prediction, using default parameters, for full-length human FUZ protein (Wang *et al.*, 2017). A triplicated contact pattern is observed, labelled F1, F2 and F3.

Supplementary Figure S6. INTU contact-map.

RaptorX coevolution-based contact prediction, using default parameters, for human INTU amino acids 271-942 protein sequence (Wang *et al.*, 2017). A triplicated contact pattern is observed, labelled I1, I2 and I3.

Supplementary Figure S7. HPS1 contact-map.

RaptorX coevolution-based contact prediction, using default parameters, for full-length human HPS1 protein (Wang *et al.*, 2017).

Supplementary Figure S8. HPS4 contact-map.

RaptorX coevolution-based contact prediction, using default parameters, for full-length human HPS4 protein (Wang *et al.*, 2017).

Supplementary Figure S9. Second Longin domain of INTU/CCZ1/HPS4.

Details are provided in the legend to Supplementary Figure S1.

Supplementary Figure S10. Second Longin domain of FUZ/MON1/HPS1.

Details are provided in the legend to Supplementary Figure S1. Numbering of Q86VX9_MON1A_HUMAN protein corresponds to its isoform 5.

Supplementary Figure S11. Third Longin domain of INTU/CCZ1/HPS4.

Details are provided in the legend to Supplementary Figure S1.

Supplementary Figure S12. Third Longin domain of FUZ/MON1/HPS1.

Details are provided in the legend to Supplementary Figure S1. Numbering of Q86VX9_MON1A_HUMAN protein corresponds to its isoform 5.
