# Supplementary figures and images for: Hexa-Longin domain scaffolds for inter-Rab signalling
Source: Bioinformatics. 2019 Sep 28;36(4):990–3. doi: 10.1093/bioinformatics/btz739 (PMC7703760; doi:10.1093/bioinformatics/btz739)

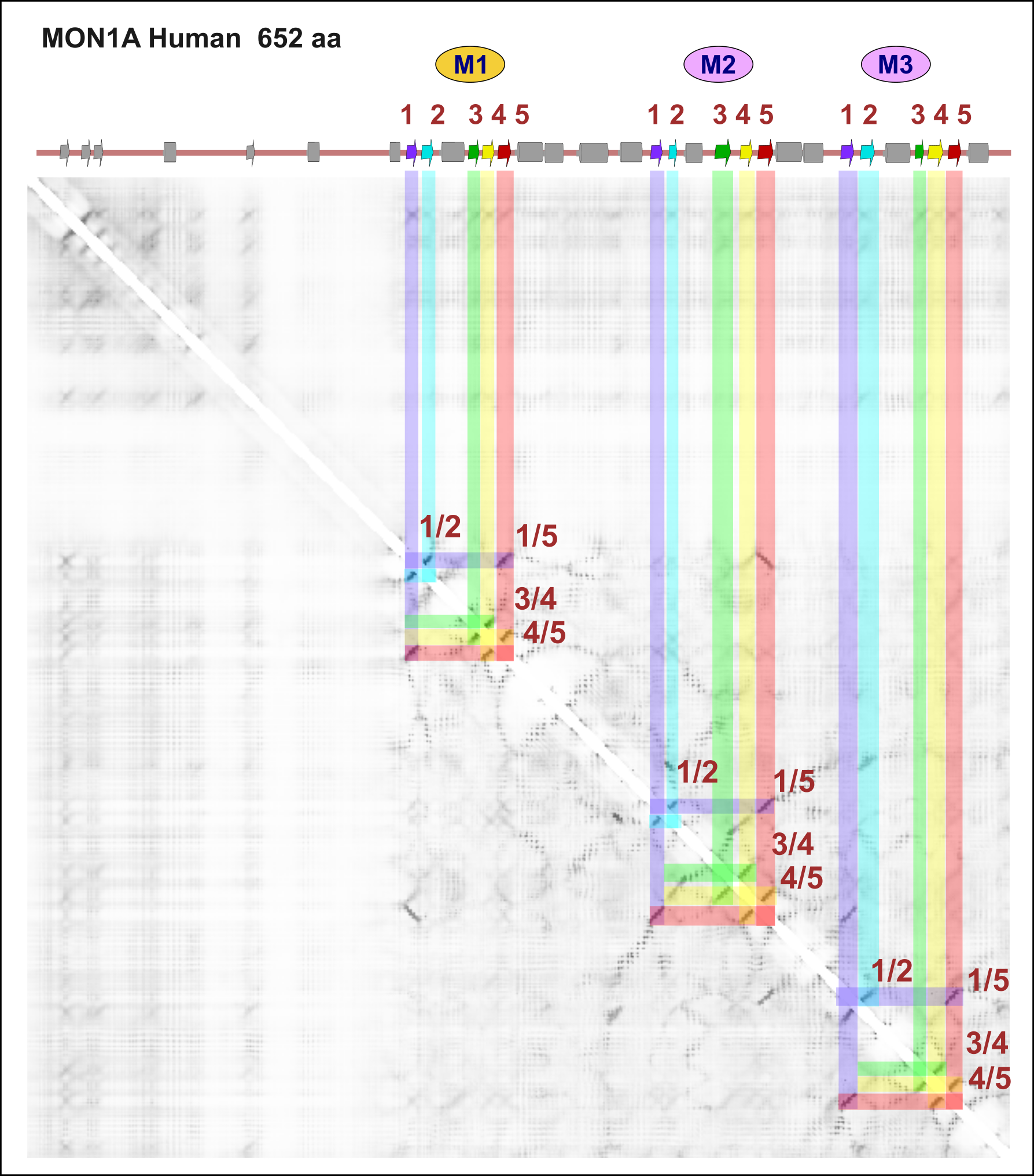

Supplement: btz739_Supplementary_Data [file btz739_supplementary_data.zip › Supp3.png]

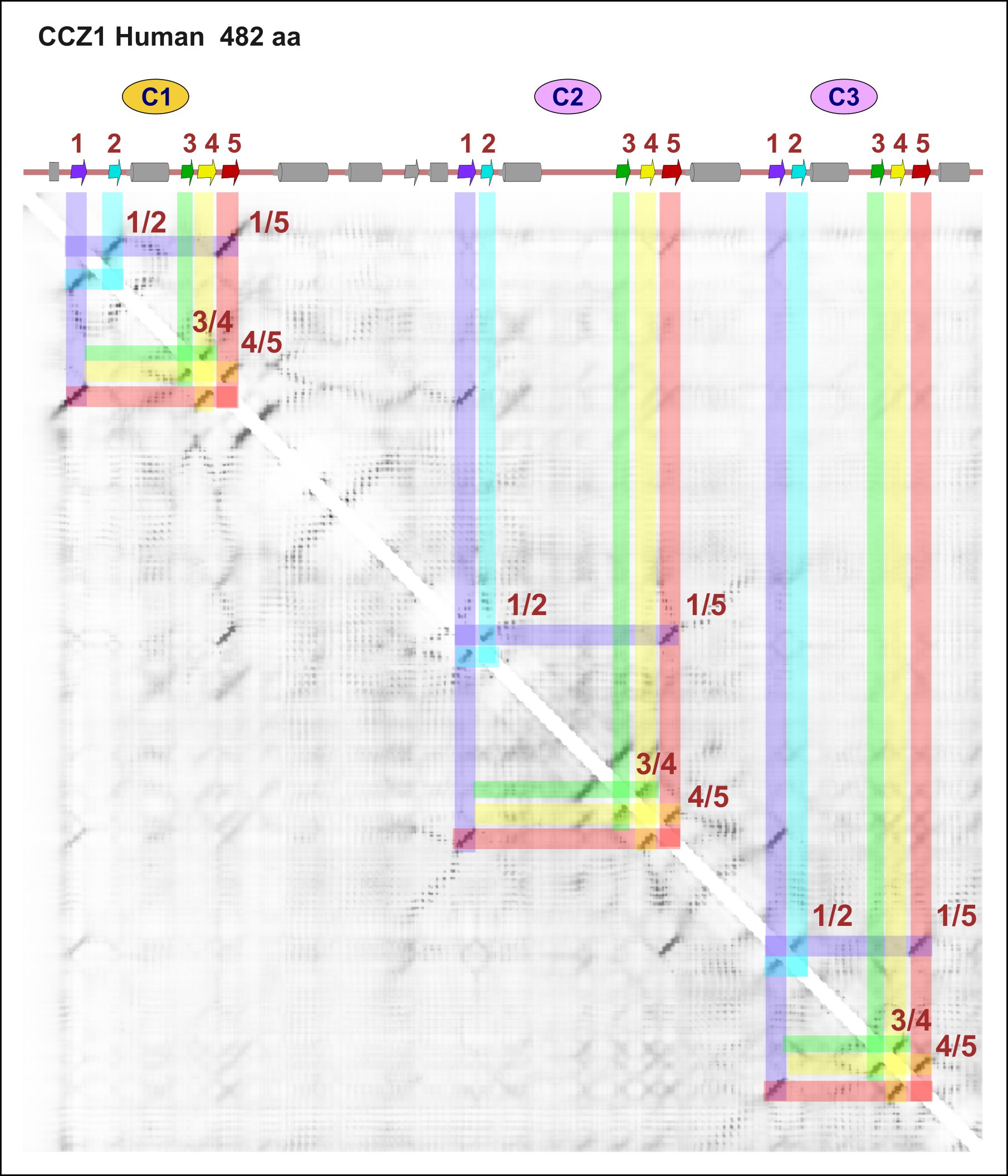

Supplement: btz739_Supplementary_Data [file btz739_supplementary_data.zip › Supp4.png]

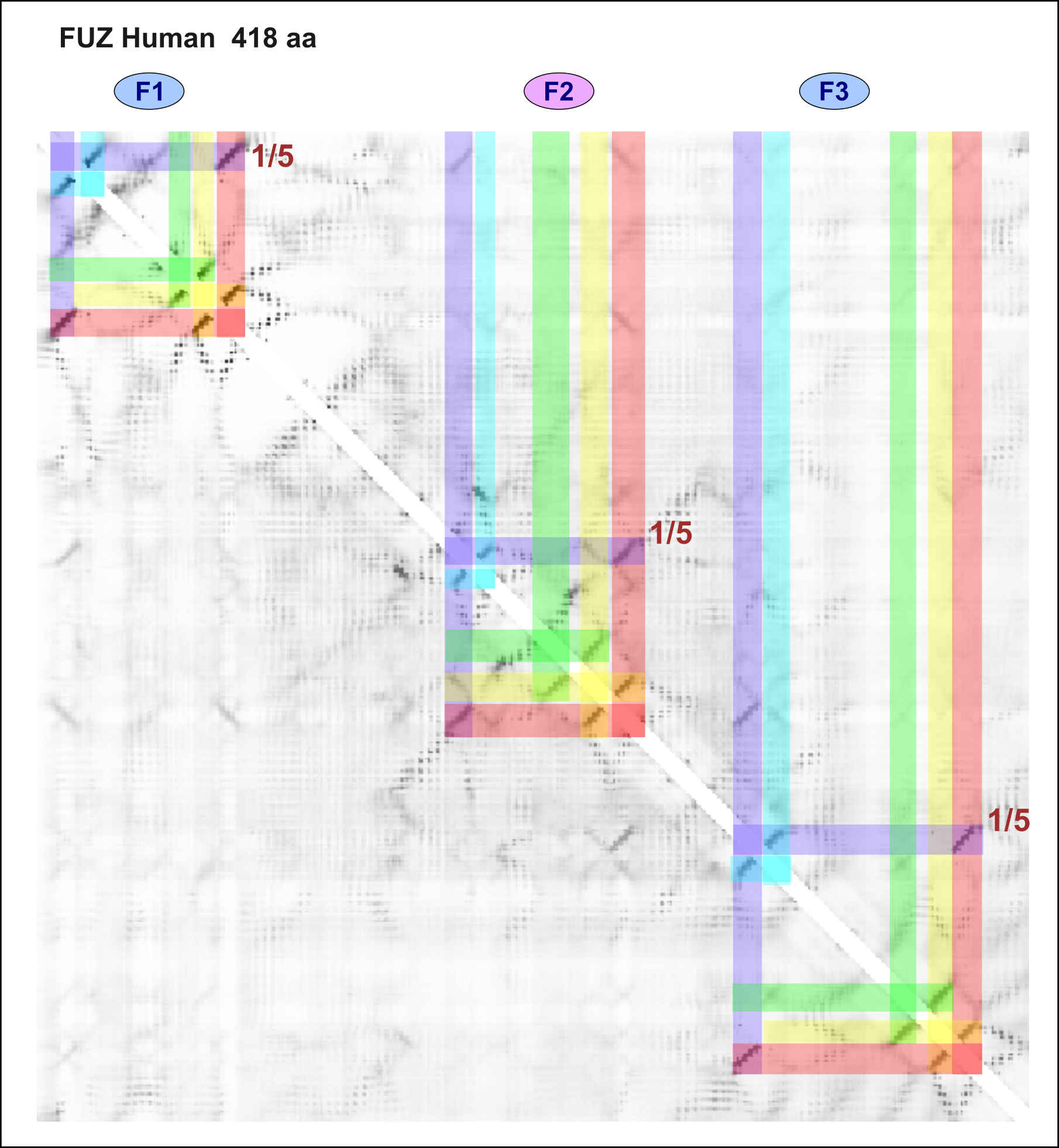

Supplement: btz739_Supplementary_Data [file btz739_supplementary_data.zip › Supp5.png]

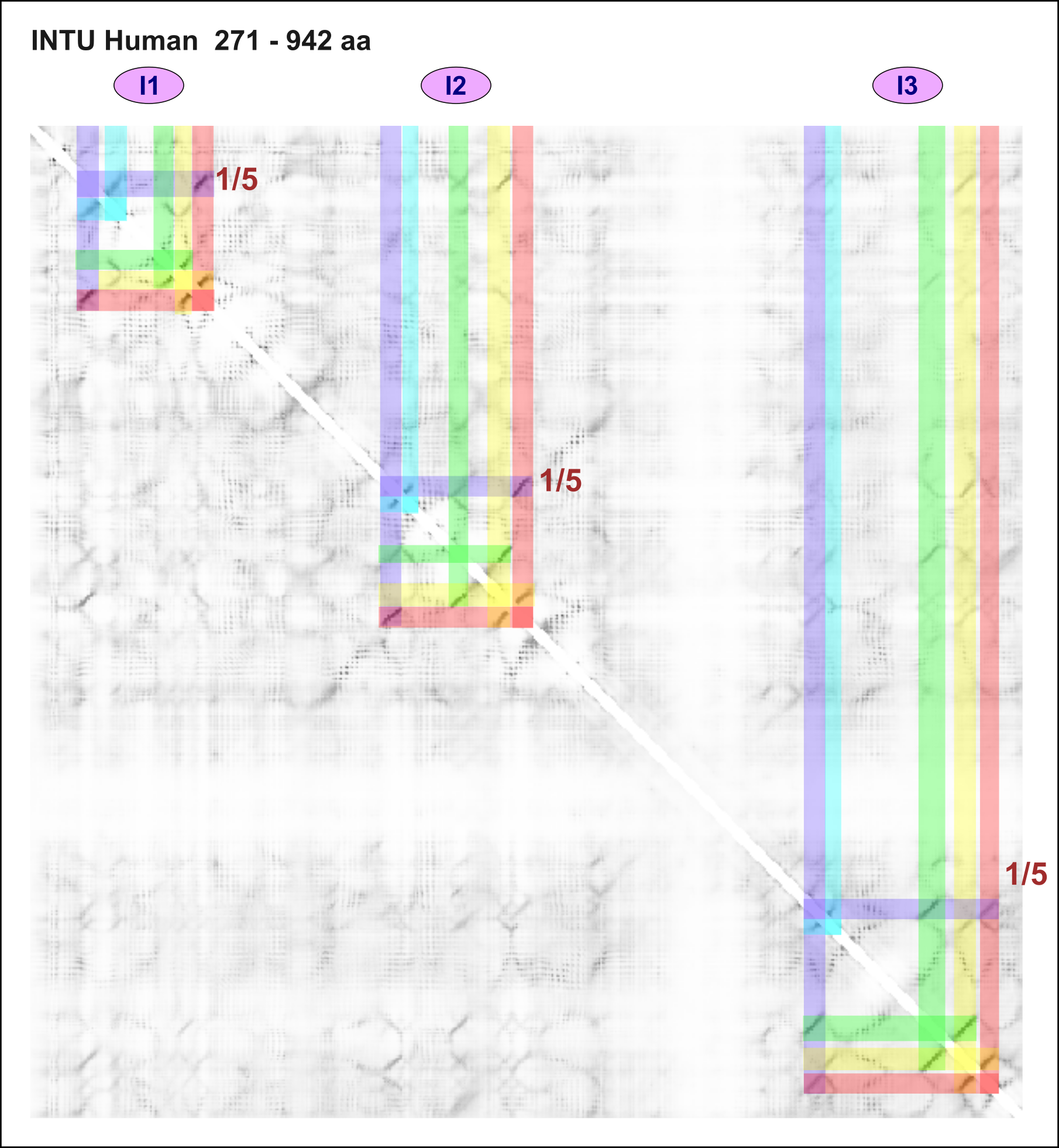

Supplement: btz739_Supplementary_Data [file btz739_supplementary_data.zip › Supp6.png]

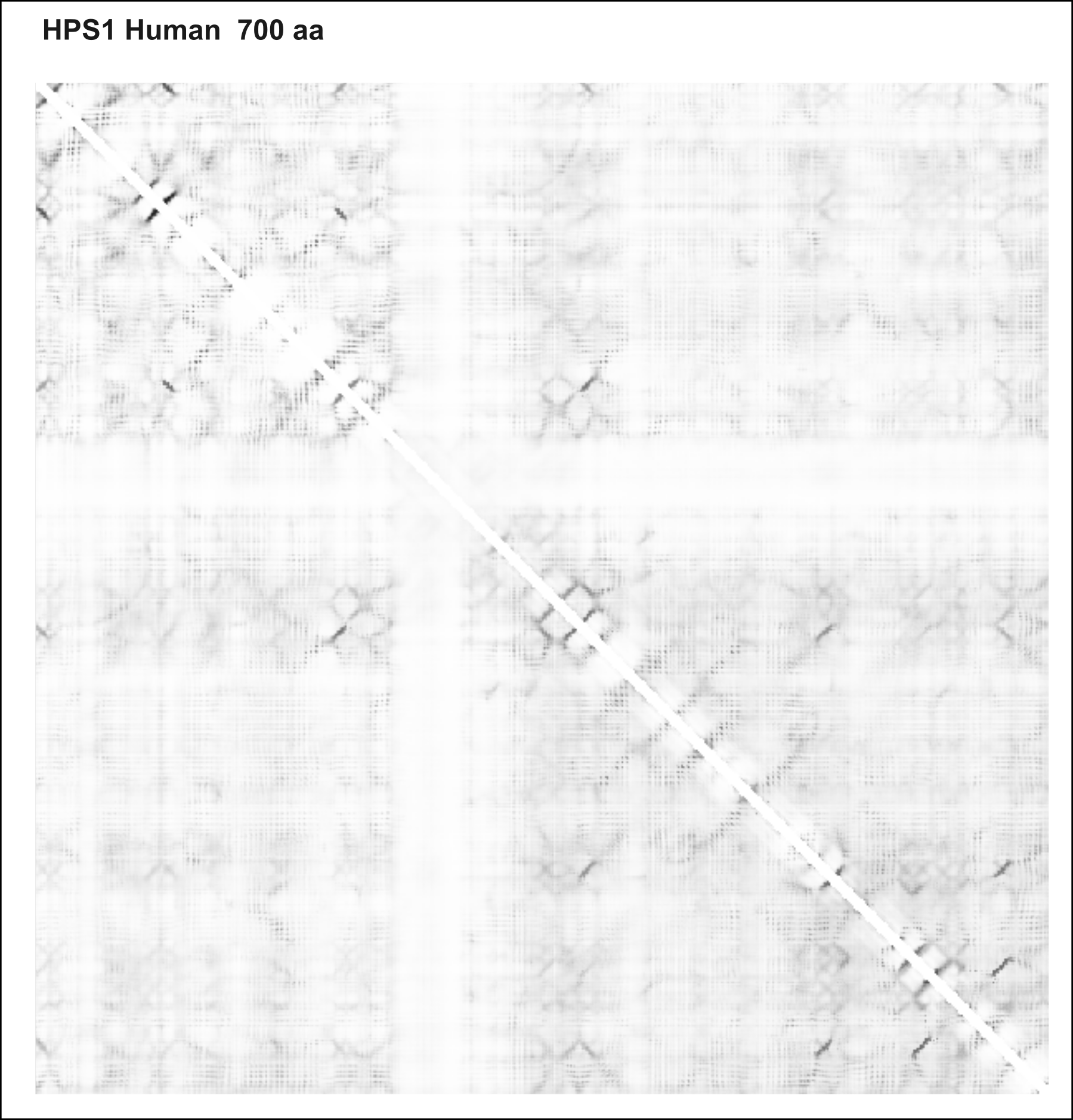

Supplement: btz739_Supplementary_Data [file btz739_supplementary_data.zip › Supp7.png]

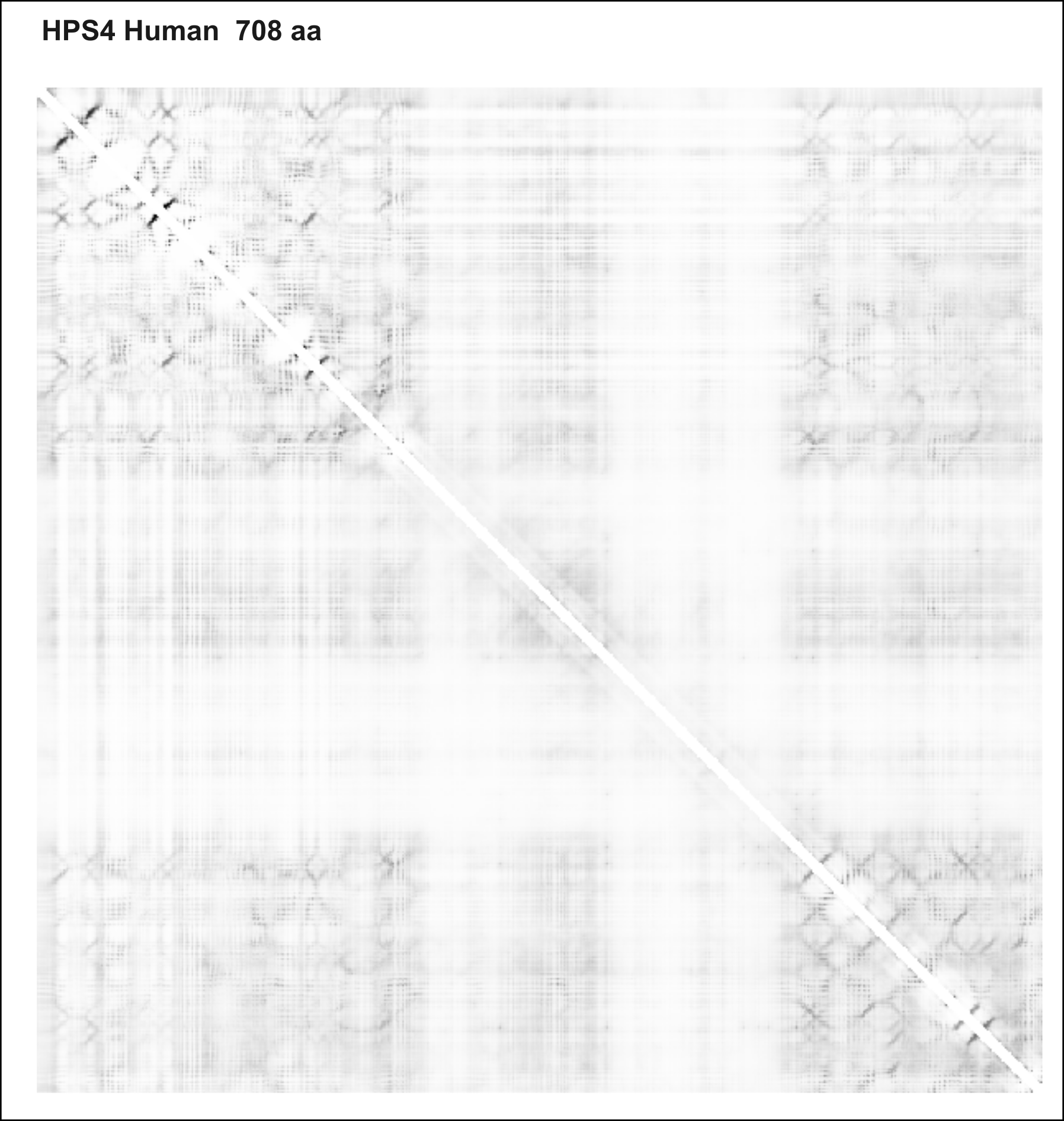

Supplement: btz739_Supplementary_Data [file btz739_supplementary_data.zip › Supp8.png]

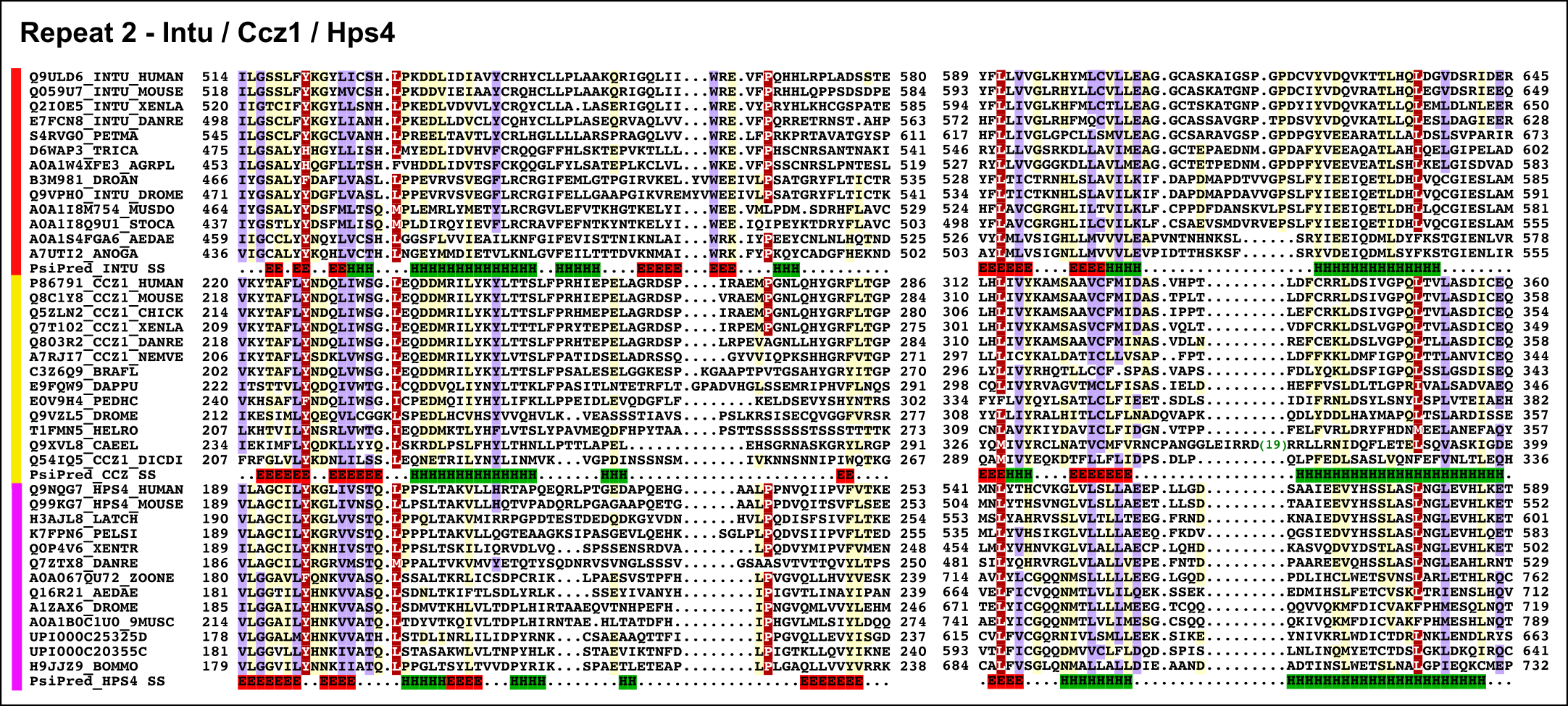

Supplement: btz739_Supplementary_Data [file btz739_supplementary_data.zip › Supp9.png]

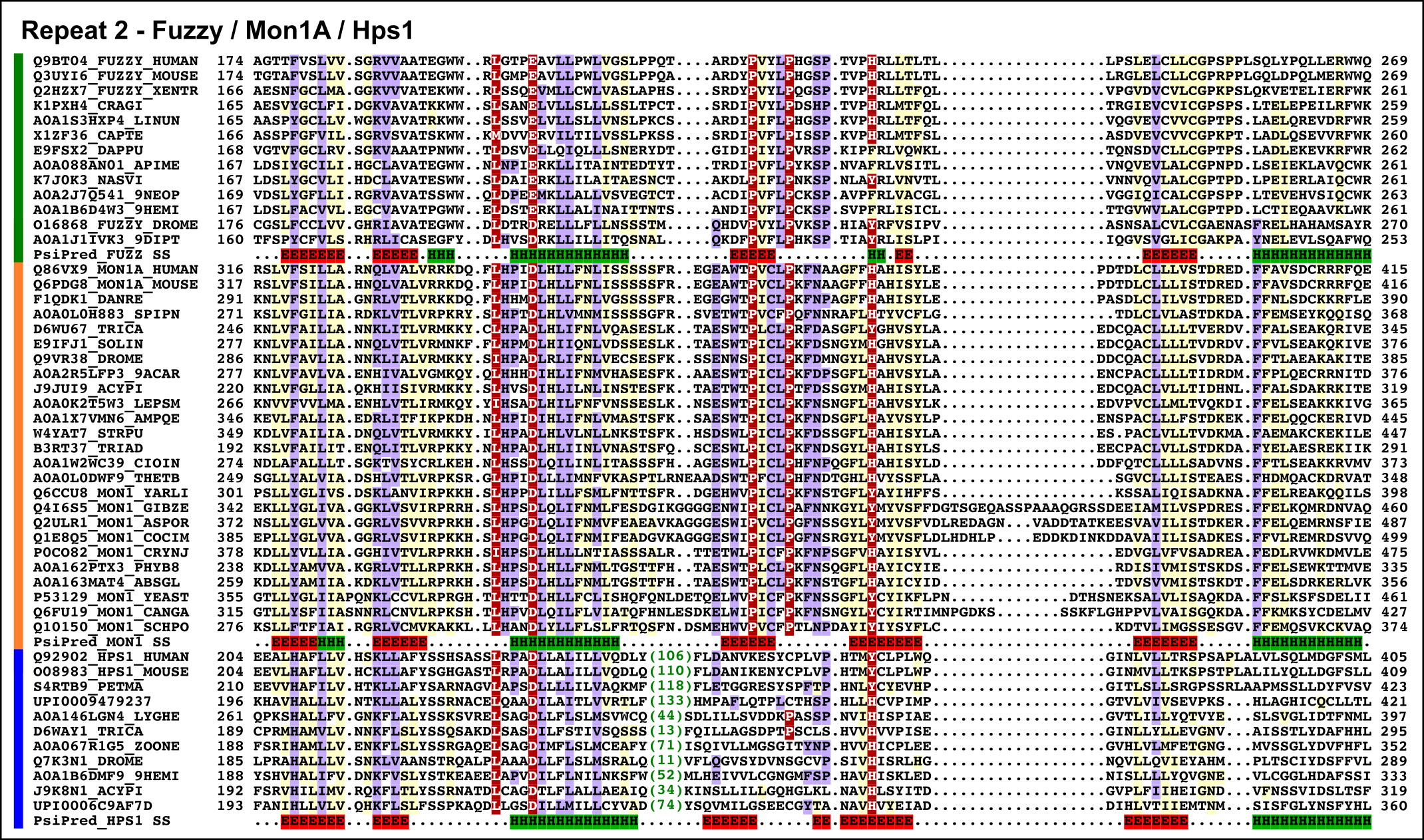

Supplement: btz739_Supplementary_Data [file btz739_supplementary_data.zip › Supp10.png]

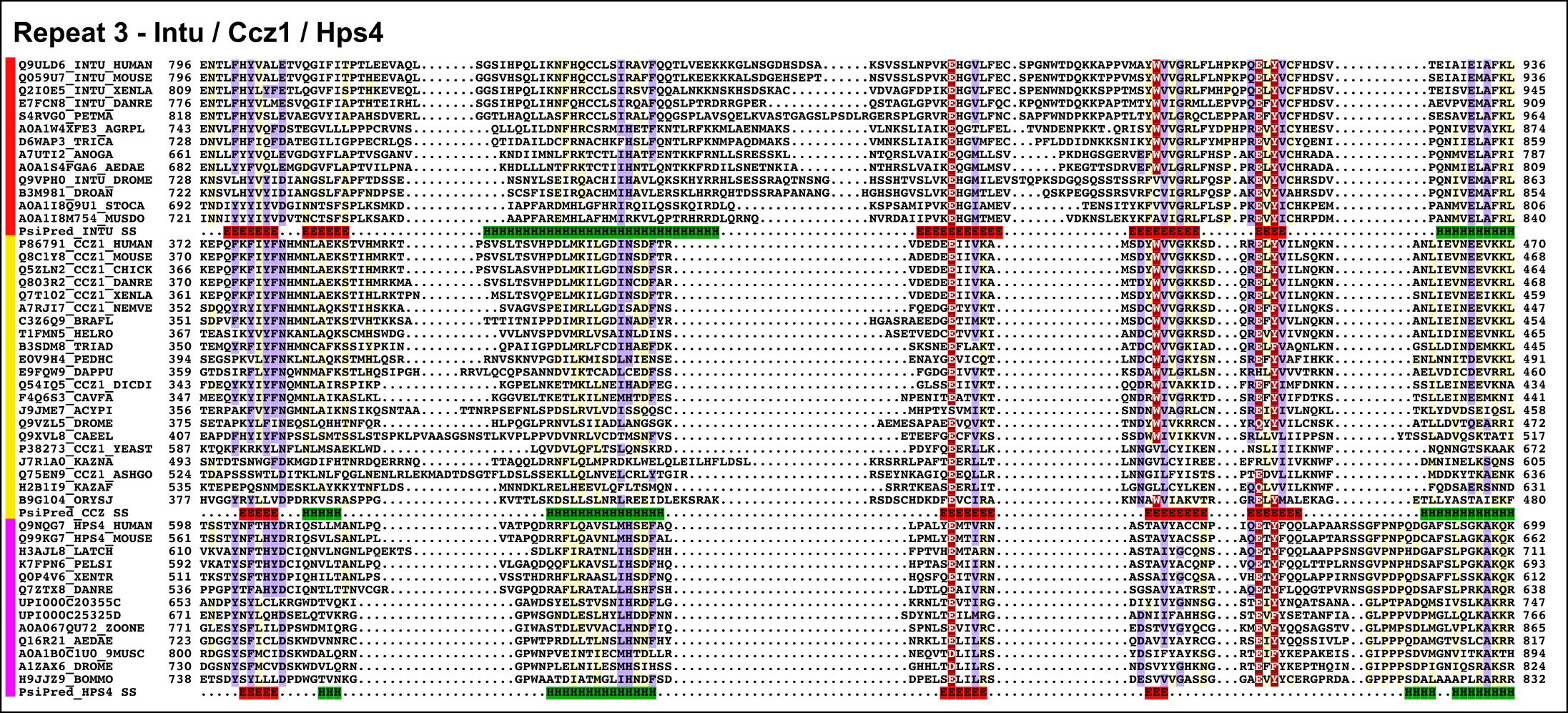

Supplement: btz739_Supplementary_Data [file btz739_supplementary_data.zip › Supp11.png]

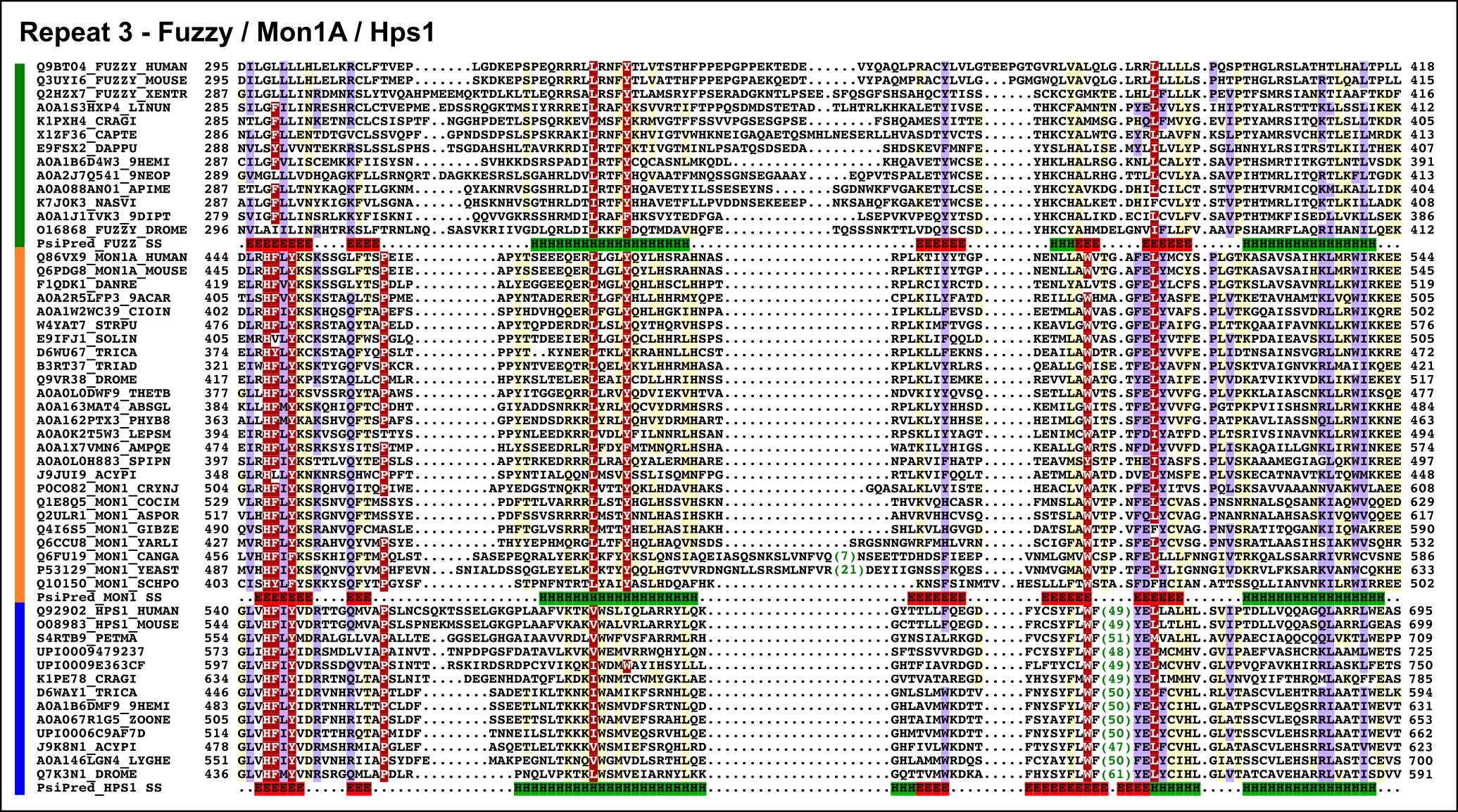

Supplement: btz739_Supplementary_Data [file btz739_supplementary_data.zip › Supp12.png]

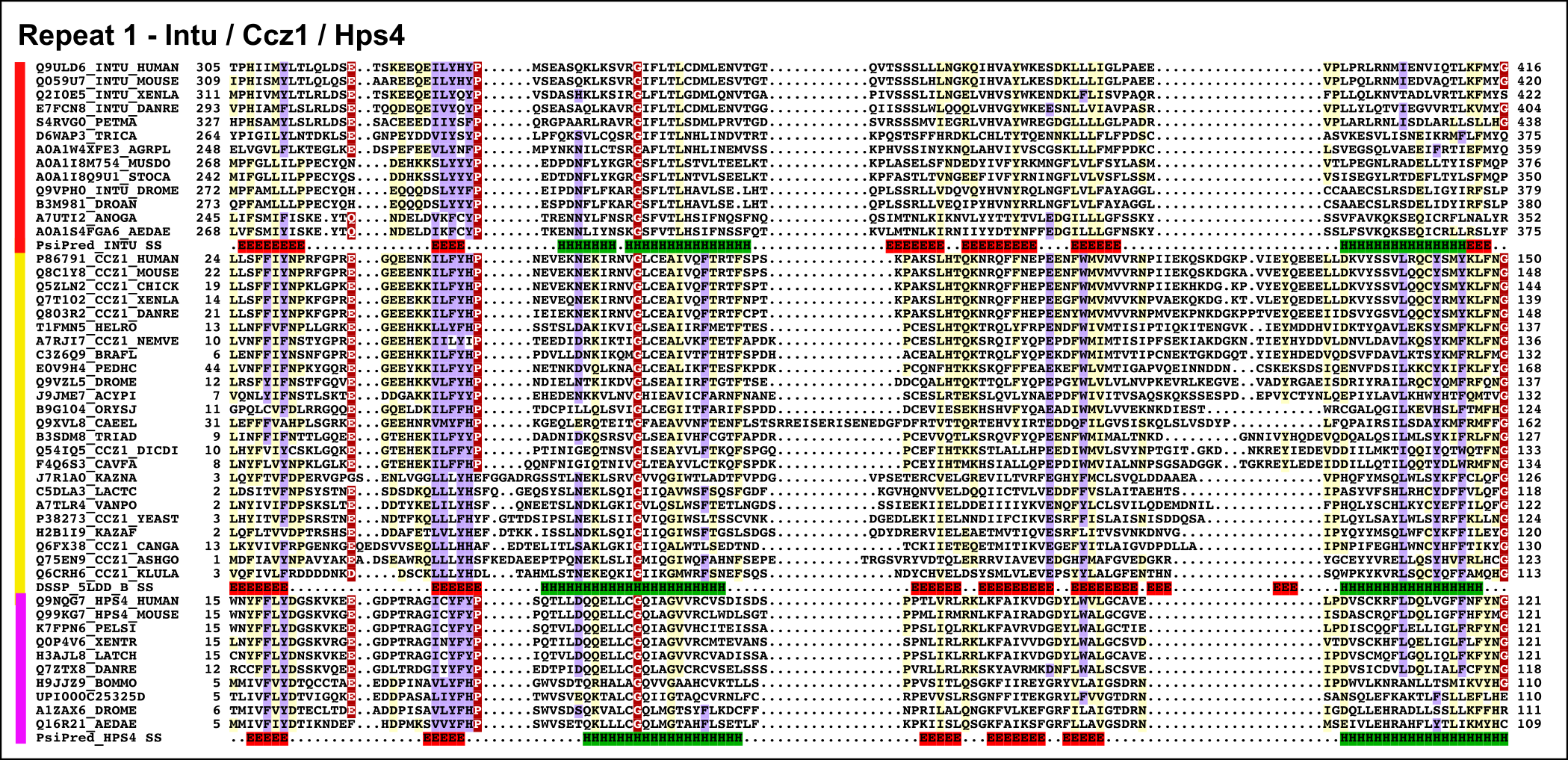

Supplement: btz739_Supplementary_Data [file btz739_supplementary_data.zip › Supp1.png]

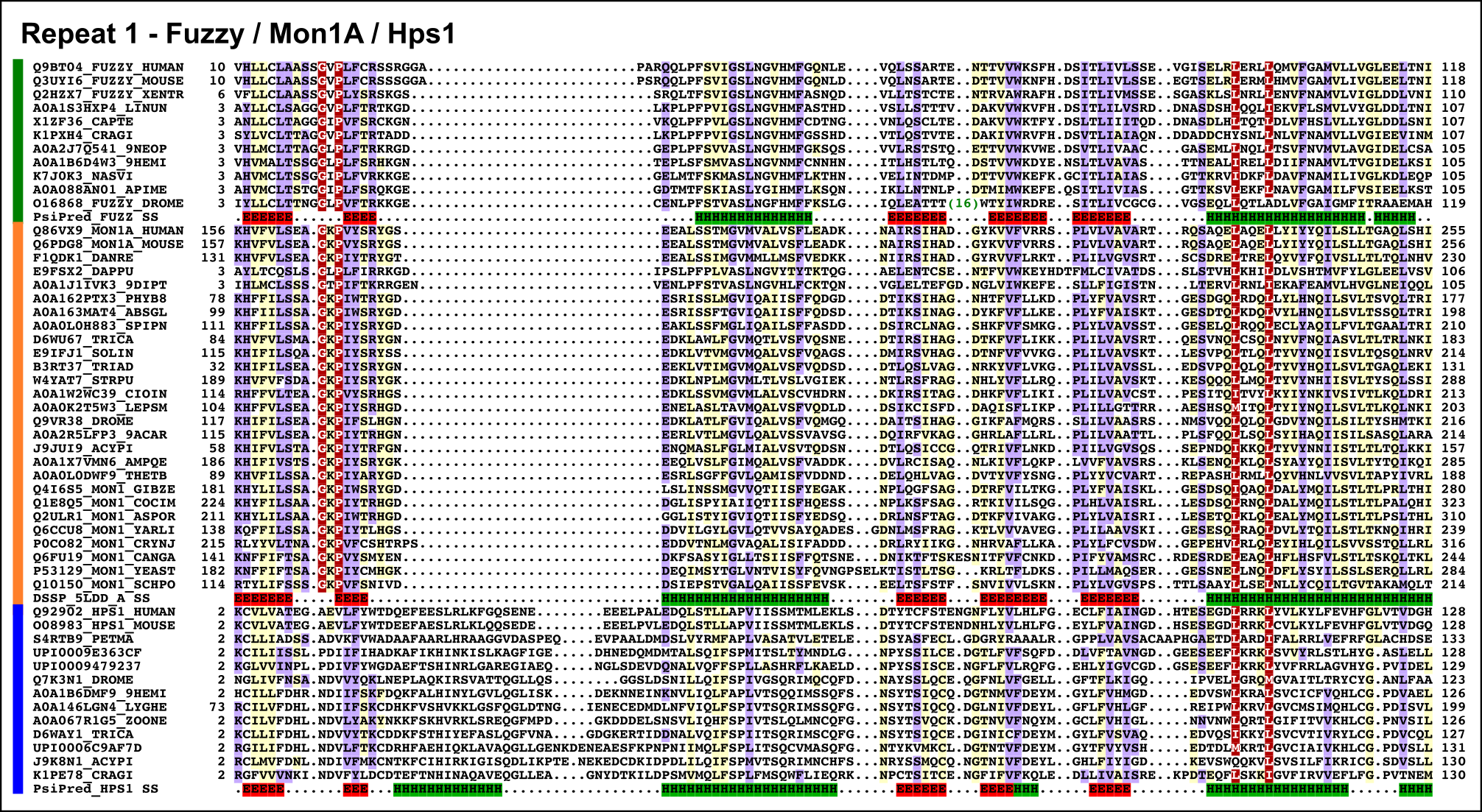

Supplement: btz739_Supplementary_Data [file btz739_supplementary_data.zip › Supp2.png]
